# Synthesis and Bioactivity of *N*-Benzoyl-*N*′-[5-(2′-substituted phenyl)-2-furoyl] Semicarbazide Derivatives

**DOI:** 10.3390/molecules15064267

**Published:** 2010-06-11

**Authors:** Zining Cui, Yun Ling, Baoju Li, Yongqiang Li, Changhui Rui, Jingrong Cui, Yanxia Shi, Xinling Yang

**Affiliations:** 1 Key Laboratory of Pesticide Chemistry and Application, Ministry of Agriculture, Department of Applied Chemistry, College of Science, China Agricultural University, Beijing 100193, China; E-Mails: ziningcui@gmail.com (Z.C.); lyun@cau.edu.cn (Y.L.); lyq@nankai.edu.cn (Y.L.); chrui@ippcaas.cn (C.R.); 2 Institute of Vegetables and Flowers, Chinese Academy of Agricultural Science, Beijing 100081, China; E-Mails: libaoju62197975@126.com (B.L.); shiyanxia813@163.com (Y.S.); 3 State Key Laboratory of Natural and Biomimetic Drugs, School of Pharmaceutical Science, Peking University, Beijing 100083, China; E-Mail: jrcui@bjmu.edu.cn (J.C.)

**Keywords:** chitin synthesis inhibitors, benzoylphenylurea, semicarbazide, synthesis, bioactivity

## Abstract

In order to find novel chitin synthesis inhibitors (CSIs) with good activity, benzoylphenylurea, a typical kind of CSIs, was chosen as the lead compound and 15 novel derivatives containing furan moieties were designed by converting the urea linkage of benzoylphenylureas into a semicarbazide and changing the aniline part into furoyl groups. The title compounds were synthesized by the reaction of substituted benzoyl isocyanates with 5-(substituted phenyl)-2-furoyl hydrazine, and the structures were confirmed by IR, ^1^H-NMR, elemental analysis and single crystal X-ray diffraction analyses (compound **E2**). The bioassay results indicated that the title compounds exhibit good insecticidal activity, especially towards *Plutella xylostella* L., but had lower fungicidal activity. Inspiringly, the title compounds possessed obvious anticancer activity against human promyelocytic leukemic cell line (HL-60), and some of the title compounds also had activity against human hepatocellular carcinoma cell line (Bel-7402), human gastric carcinoma cell line (BGC-823), and human nasopharyngeal carcinoma cell line (KB). The results indicated that the linkage in the lead compounds was important to the bioactivity and spectra. The modification on the urea linkage is an effective strategy to discover new pesticide and drug candidates.

## 1. Introduction

Chitin, the *β*-(1→4) glycoside polymer of *N*-acetyl-D-glucosamine, is a major structural component of insect cuticles. In addition to the insect cuticles, chitin is also present in cell walls of fungi and protozoa, but is absent in higher plants and vertebrates. This species-specificity provides the rationale to select chitin as a safe target for pest control agents [1,2]. Chitin synthesis inhibitors (CSIs), which can interfere with chitin formation, will affect the normal growth and development process of insects and fungi but have no effect on plants and vertebrates. The unique mode of action coupled with excellent activity on target and lower toxicity to non-target organisms (including many beneficial arthropods) made these compounds a new tool for integrated pest management (IPM) [3,4,5,6,7,8,9,10,11,12,13,14,15].

**Scheme 1:**
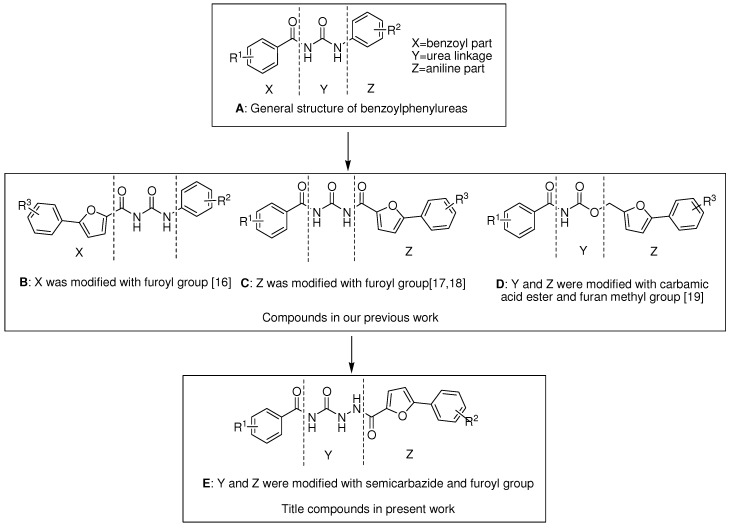
Design strategy for title compounds **E**.

Benzoylphenylureas (BPUs) have been developed as a typical kind of chitin synthesis inhibitors since Dimilin (diflubenzuron) was introduced into market in the 1970s [3,4]. Their general structures **A** (Scheme 1) consist of three parts including the benzoyl (X), the urea linkage (Y), and the aniline (Z) [7]. 

Numerous modifications on the benzoyl (X) and aniline (Z) parts have resulted in the discovery of many commercial insecticides, such as chlorfluazuron, teflubenzuron, bistrifluoron, and noviflumuron [7,8,9,10,11]. However, modification on the urea linkage (Y) has been considered less, although some efforts on this part have been made. For example, Grosscurt found that the compounds had good insecticidal properties when the urea linkages were converted into carbamoyl-2-pyrazolines [12]. Wang’s group reported that the compounds resulting from substitution of the hydrogen on the nitrogen atom of the urea linkage with carbamylosulfenyl or carbamate groups could retain the insecticidal activity [13,14,15].

In our previous work, the benzoyl (X) part was modified with a furoyl group to get benzoylureas B [16], and the aniline (Z) part was replaced with a furoyl group to obtain dibenzoylureas C [17,18]. The bioassay results indicated that some of the resulting compounds had certain insecticidal activity, whereas when the Y and Z moieties were modified with carbamic acid ester and furan methyl groups, the insecticidal activity disappeared and the compounds exhibited good fungicidal activity [19]. In the present work, an effort of structure optimization was carried out, in which the urea linkage was converted to a semicarbazide, while the aniline part was replaced with a 5-phenyl-2-furoyl group (Scheme 1). Fifteen novel *N*-benzoyl-*N*′-[5-(2′-substituted phenyl)-2-furoyl] semicarbazides (**E**) were designed and synthesized for the purpose of new pesticide and anticancer drug discovery. Their *in vivo* insecticidal activity and *in vitro* fungicidal and antitumor activities were preliminarily evaluated.

## 2. Results and Discussion

### 2.1. Chemistry

**Scheme 2:**
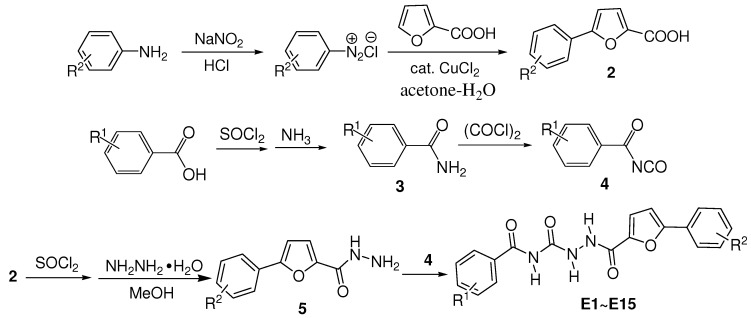
General synthetic procedure for title compounds **E**.

The synthetic route of compounds **E** is shown in Scheme 2, where the starting materials were substituted anilines and benzoic acids. All of the title compounds were prepared by the nucleophilic addition reaction of 5-substituted aryl-2-furoyl hydrazine with benzoyl isocyanate under an anhydrous atmosphere.

All of the structures of the title compounds were confirmed by ^1^H-NMR, IR, and elemental analyses. In the IR spectra, the compounds showed absorption bands around 3,500 and 3,200 cm^-1^ originating from the N-H stretching vibration. The strong bands around 1,750 cm^-1^ could be assigned to the C=O stretching vibration. The bands between 1,690 and 1,650 cm^-1^ were carbonyl vibration of the secondary amide. Absorption bands around 1,610, 1,520 and 1,480 cm^-1^ were attributed to the frame vibrations of the phenyl and furan rings.

In the ^1^H-NMR spectrum, one sharp peak in the range from 9.70 to 11.50 ppm was due to the presence of NH. The splits of most compounds were normal, except for the compounds with fluorine substitution because of the coupling and splitting between fluorine and hydrogen. The fluorine atom splits a hydrogen proton into a doublet, which complicated the proton signals. The protons on phenyl rings were mostly split into multiple peaks in the range from 7.20 to 8.10 ppm and the protons on the furan ring were split into a doublet in the range from 6.90 to 7.40 ppm.

**Figure 1:**
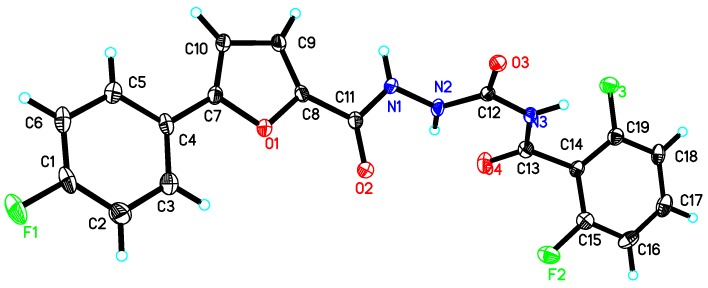
Molecular structure of compound **E2**, showing 30% probability ellipsoids, H atoms were shown as small spheres of arbitrary radii.

The structures were further conformed by single crystal X-ray analysis of a typical example, compound **E**2. Figure 1 gives a perspective view of the compound sad the crystal data are presented in Table 1. Some important bond lengths, angels, and torsion angles of compound **E2** are given in Table 2. It could be seen from the X-ray analysis of **E2** that the single bonds distances of C11-N1, C12-N2, C13-N3, and C12-N3 (1.363(3), 1.342(3), 1.369(3), and 1.398(3) Å) are equal to the van der Waals’ radii for a C–N double bond (1.35 Å), the single bonds C4-C7, C8-C11, and C13-C14 (1.461(4), 1.470(4), and 1.504(4) Å) are shorter than the standard C–C single bond (1.54 Å), but longer than C–C double bond (1.34 Å), and the N1–N2 (1.387(3) Å) single bond is shorter than the standard N–N single bond (1.45 Å), but longer than a N–N double bond (1.25 Å). All of these clearly indicated that the *p* orbital of N atoms conjugated with the π molecular orbital and formed the delocalized π-bonds with the conjoint furan and benzene ring. Unexpectedly, the *p* orbitals of N1, N2, and N3 seemed not to be conjugated with the π molecular orbital of the C11–O2, C12–O3, and C13-O4 double bonds, which was explained by the bond length of C11–O2, C12-O3, and C13–O4 (1.228(3), 1.224(3), and 1.219(3) Å) that followed in the normal range of C–O double bonds (1.19-1.23 Å).

In the crystal of the structure, C(1), C(2), C(3), C(4), C(5), and C(6) formed a plane with a mean deviation of 0.0099 Å, defined as plane I; C(7), C(8), C(9), C(10), and O(1) formed a plane with the a mean deviation of 0.0009 Å, defined as plane II; O(2), C(11), N(1), and N(2) were nearly coplanar with a mean deviation of 0.0144 Å, defined as plane III; O(3), C(12), N(3), C(31) and O(4) were nearly coplanar with a mean deviation of 0.0163 Å, defined as plane IV; C(14), C(15), C(16), C(17), C(18), and C(9) formed a plane with a mean deviation of 0.0075 Å, defined as plane V (Figure 1). Plane II, plane III, plane IV and plane V form a dihedral angle with plane I of 4.2, 3.3, 73.3, and 17.5°. Plane III, plane IV and plane V form a dihedral angle with plane II of 7.2, 75.8, and 19.9°. Plane IV and plane V form a dihedral angle with plane III of 70.1 and 14.5. And the dihedral angle between plane IV and plane V is 55.9°. The related data are summarized in Table 3.

### 2.2. Biological activity

Considering that the lead compounds **B** and **C** had good insecticidal activity against dipterous and lepidopterous insects, and **D** had good fungicidal activity, we chose five strains of fungi and three kinds of insects to evaluate whether the designed compounds retained the insecticidal and fungicidal activity.

The bioassay showed that the title compounds exhibited lower fungicidal activity *in vivo* against the five strains of fungi at the concentration of 50 μg mL^-1^ (Table 4). In the insecticidal activity shown in Table 5, some title compounds possessed good activity to the *Plutella xylostella* L. at the concentration of 500 μg mL^-1^, such as **E10** and **E15** were 95.0% and 85.0%, respectively.

All the title compounds exhibited lower *in vitro* insecticidal activity towards *Aphis gossypii* at the concentration of 500 μg mL^-1^. The highest mortality was 38.0% of **E13**. The commercial pesticides RH-5849 and hexaflumuron, which display similar structures as the title compounds also showed lower activity to *Aphis gossypii* (the results were 22.1% and 22.6%), which indicated that these kinds of structures were not suitable for the control of *Aphis gossypii*. None of the title compounds had activity towards *Tetranchus urticae* at the concentration of 500 μg mL^-1^.

Ren [20] and Nishat [21] reported that compounds containing semicarbazides showed excellent anticancer activity. Enlightened by the results from Ren and Nishat, we also studied the anticancer activity of the title compounds, in which the urea linkage was converted into a semicarbazide.

Four kinds of cancer cells lines, human promyelocytic leukemic cell line (HL-60), human hepatocellular carcinoma cell line (Bel-7402), human gastric carcinoma cell line (BGC-823), and human nasopharyngeal carcinoma cell line (KB) were chosen for the bioassay. As shown in Table 6, most of the compounds **E** showed good activity against HL-60. Moreover, some of them exhibited good activity to the solid tumor cells BGC-823 and Bel-7402. Among them **E15** exhibited higher activity and broader spectra than others. It had excellent activity of 0.14, 0.36 and 0.09 mM against HL-60, Bel-7402, and BGC-823, respectively.

Comparing the title compounds E with lead compounds B, C, and D, it can be seen that the linkage (Y) is important for the bioactivity and spectra. When the linkage was urea and semicarbazide, the compounds exhibited insecticidal activity, especially towards lepidopterous insects, but had lower effects on fungi. When the linkage was converted to a carbamic acid ester, the compounds had no insecticidal activity, but possessed good fungicidal activity. Moreover, the title compounds E exhibited anticancer activity.

## 3. Experimental

### 3.1. Materials and methods

All the melting points were determined with a Cole-Parmer melting point apparatus and are uncorrected. IR spectra were recorded on a Thermo Nicolet Nexus 470 FT-IR spectrometer as KBr pellets. ^1^H-NMR spectra were recorded on a Bruker DPX300 instrument (Switzerland), with DMSO-*d_6_* as solvent and tetramethylsilane as the internal standard. Elemental analysis was performed by the Analytical Center in the Institute of Chemistry, Chinese Academy of Sciences, using a Flash EA 1112 elemental analyzer. The solvents and reagents were mainly purchased from the Beijing Chemical Reagents Co., P. R. China. 

### 3.2. General procedure for the preparation of key intermediates **2** and **4**

The different substituted benzoyl isocyanates **4** were obtained by the reaction of the corresponding benzamides and oxalyl dichloride according to the literature [22]. The 5-substituted phenyl-2-furancarboxylic acids **2** were prepared according to the method given in reference [23].

### 3.3. General synthetic procedure for compounds **E**

According to the literature [23], different 5-(substituted phenyl)-2-furoyl hydrazines **5** were prepared. Then **4** (5 mmol) was added to a solution of compound **5** (5 mmol) in purified toluene (25 mL). The reaction mixture was kept at room temperature for 2 h. The solvent were evaporated off under reduced pressure, and the resulting solid was purified by vacuum column chromatography on silica gel using petroleum ether (60–90 °C) and ethyl acetate as the eluent to yield **E**.

*N-benzoyl-N′-[5-(4′-chlorophenyl)-2-furoyl] semicarbazide* (**E1**): White powdery crystals: yield 78.9%, m.p. 238–239 °C. ^1^H-NMR *δ*: 7.22 (d, *J* = 3.57 Hz, 1H, FuH), 7.34 (d, *J* = 3.63 Hz, 1H, FuH), 7.52–7.72 (m, 5H, 2ArH-Fu + 3ArH), 7.92–8.04 (m, 4H, 2ArH-Fu + 2ArH), 10.13 (s, 1H, NHCO), 10.71 (s, 1H, CONH), 11.11 (s, 1H, CONHCO); IR *ν*: 3405.9, 3255.7, 1686.8, 1653.8, 1604.8, 1534.9, 1474.4, 1441.7, 1271.5, 1093.0, 1025.6 cm^-1^; Anal. calcd.(%) for C_19_H_14_ClN_3_O_4_: C 59.46, H 3.68, N 10.95; found C 59.73, H 3.58, N 10.89.

*N-2,6-difluorobenzoyl-N′-[5-(4′-fluorophenyl)-2-furoyl] semicarbazide* (**E2**): Light yellow powdery crystals: yield 75.8%, m.p. 207–208 °C. ^1^H-NMR *δ*: 7.15 (d, *J* = 3.60 Hz, 1H, FuH), 7.23–7.40 (m, 5H, FuH + 2ArH-Fu + 2ArH), 7.57–7.67 (m, 1H, ArH), 7.99–8.06 (m, 2H, ArH-Fu), 9.72 (s, 1H, NHCO), 10.63 (s, 1H, CONH), 11.48 (s, 1H, CONHCO); IR *ν*: 3425.0, 3262.8, 3131.9, 2951.2, 1738.0, 1698.2, 1648.5, 1626.0, 1592.9, 1512.0, 1482.2, 1315.9, 1284.2, 1265.3, 1234.1, 1161.2, 1109.4, 1030.2, 1014.6 cm^-1^; Anal. calcd.(%) for C_19_H_12_F_3_N_3_O_4_: C 56.58, H 3.00, N 10.42; found C 56.53, H 3.08, N 10.37.

*N-3-methylbenzoyl-N′-[5-(4′-chlorophenyl)-2-furoyl] semicarbazide* (**E3**): Light yellow powdery crystals: yield 83.6%, m.p. 221–222 °C. ^1^H-NMR *δ*: 2.39 (s, 3H, CH_3_), 7.23 (d, *J* = 3.57 Hz, 1H, FuH), 7.34 (d, *J* = 3.66 Hz, 1H, FuH), 7.43–7.47 (m, 2H, ArH), 7.58 (dd, *J* = 6.71, 1.94 Hz, 2H, ArH-Fu), 7.80–7.86 (m, 2H, ArH), 7.99–8.02 (m, 2H, ArH-Fu), 10.13 (s, 1H, NHCO), 10.72 (d, *J* = 1.08 Hz, 1H, CONH), 11.05 (s, 1H, CONHCO); IR *ν*: 33401.9, 3245.5, 3131.9, 1712.5, 1669.7, 1586.1, 1538.4, 1510.3, 1473.8, 1410.8, 1318.8, 1278.0, 1234.9, 1095.5, 1034.1 cm^-1^; Anal. calcd.(%) for C_19_H_14_ClN_3_O_4_: C 60.38, H 4.05, N 10.56; found C 60.35, H 4.20, N 10.47.

*N-2,6-difluorobenzoyl-N′-[5-(4′-chlororophenyl)-2-furoyl] semicarbazide* (**E4**): Light brown powdery crystals: yield 82.6%, m.p. 220–221 °C. ^1^H-NMR *δ*: 7.22–7.28 (m, 3H, FuH + 2ArH), 7.34 (d, *J* = 3.66 Hz, 1H, FuH), 7.56–7.72 (m, 3H, 2ArH-Fu + ArH), 7.98–8.02 (m, 2H, ArH-Fu), 9.74 (s, 1H, NHCO), 10.67 (s, 1H, CONH), 11.48 (s, 1H, CONHCO); IR *ν*: 3425.0, 3266.2, 3143.5, 1738.7, 1699.1, 1649.4, 1625.3, 1592.1, 1527.1, 1470.1, 1314.6, 1282.4, 1265.2, 1236.8, 1111.4, 1094.7, 1012.7 cm^-1^; Anal. calcd.(%) for C_19_H_12_ClF_2_N_3_O_4_: C 54.36, H 2.88, N 10.01; found C 54.57, H 3.10, N 10.00.

*N-2-chlorobenzoyl-N′-[5-(4′-chlorophenyl)-2-furoyl] semicarbazide* (**E5**): White powdery crystals: yield 85.1%, m.p. 196–197 °C. ^1^H-NMR *δ*: 7.22 (d, *J* = 3.60 Hz, 1H, FuH), 7.34 (d, *J* = 3.63 Hz, 1H, FuH), 7.43–7.62 (m, 6H, 2ArH-Fu + 4ArH), 7.99–8.02 (m, 2H, ArH-Fu), 9.84 (s, 1H, NHCO), 10.68 (s, 1H, CONH), 11.26 (s, 1H, CONHCO); IR *ν*: 3733.6, 3419.6, 3239.0, 1727.9, 1689.1, 1650.9, 1523.6, 1488.8, 1471.0, 1433.8, 1313.9, 1281.7, 1246.6, 1112.3, 1093.7, 1056.4, 1028.9 cm^-1^; Anal. calcd.(%) for C_19_H_13_Cl_2_N_3_O_4_: C 54.56, H 3.13, N 10.05; found C 54.23, H 3.30, N 9.86.

*N-2,6-dichlorobenzoyl-N′-[5-(4′-methylphenyl)-2-furoyl] semicarbazide* (**E6**): White powdery crystals: yield 78.6%, m.p. 210–211 °C. ^1^H-NMR *δ*: 2.35 (s, 3H, CH_3_), 7.10 (d, *J* = 3.30 Hz, 1H, FuH), 7.29–7.32 (m, 3H, 2ArH-Fu + FuH), 7.46–7.60 (m, 3H, ArH), 7.86 (d, *J* = 7.83 Hz, 2H, ArH-Fu), 9.83 (s, 1H, NHCO), 10.63 (s, 1H, CONH), 11.54 (s, 1H, CONHCO); IR *ν*: 3247.1, 3135.7, 2973.3, 1716.4, 1700.4, 1677.6, 1596.8, 1535.3, 1486.2, 1433.1, 1307.0, 1260.4, 1211.8, 1144.4, 1019.7 cm^-1^; Anal. calcd.(%) for C_20_H_15_Cl_2_N_3_O_4_: C 55.57, H 3.50, N 9.72; found C 55.61, H 3.76, N 9.67.

*N-2,6-difluorobenzoyl-N′-[5-(4′-methylphenyl)-2-furoyl] semicarbazide* (**E7**): Light yellow powdery crystals: yield 78.2%, m.p. 211–212 °C. ^1^H-NMR *δ*: 2.35 (s, 3H, CH_3_), 7.09 (d, *J* = 3.60 Hz, 1H, FuH), 7.23–7.32 (m, 5H, FuH + 2ArH-Fu + 2ArH), 7.57–7.65 (m, 1H, ArH), 7.86 (d, *J* = 8.16 Hz, 2H, ArH-Fu), 9.72 (s, 1H, NHCO), 10.59 (s, 1H, CONH), 11.47 (s, 1H, CONHCO); IR *ν*: 3276.1, 3124.8, 3023.9, 2950.5, 1739.5, 1697.2, 1650.4, 1625.5, 1593.2, 1535.1, 1468.1, 1368.1, 1316.2, 1282.0, 1233.4, 1112.2, 1058.7, 1029.8, 1012.4 cm^-1^; Anal. calcd.(%) for C_20_H_15_F_2_N_3_O_4_: C 60.15, H 3.79, N 10.52; found C 60.20, H 4.00, N 10.31.

*N-2-chlorobenzoyl-N′-[5-(4′-methylphenyl)-2-furoyl] semicarbazide* (**E8**): Light yellow powdery crystals: yield 79.5%, m.p. 206–207 °C. ^1^H-NMR *δ*: 2.35 (s, 3H, CH_3_), 7.09 (d, *J* = 3.60 Hz, 1H, FuH), 7.29–7.32 (m, 3H, 2ArH-Fu + FuH), 7.43–7.61 (m, 4H, ArH), 7.86 (d, *J* = 8.16 Hz, 2H, ArH-Fu), 9.81 (s, 1H, NHCO), 10.59 (s, 1H, CONH), 11.23 (s, 1H, CONHCO); IR *ν*: 3262.8, 3128.0, 1725.3, 1685.5, 1657.5, 1544.0, 1484.7, 1318.4, 1286.8, 1246.9, 1224.8, 1101.2, 1055.8, 1031.2 cm^-1^; Anal. calcd.(%) for C_19_H_14_ClN_3_O_4_: C 60.38, H 4.05, N 10.56; found C 60.17, H 4.09, N 10.44.

*N-2-methoxybenzoyl-N′-[5-(2′-nitrophenyl)-2-furoyl] semicarbazide* (**E9**): Brown powdery crystals: yield 73.4%, m.p. 191–192 °C. ^1^H-NMR *δ*: 3.93 (s, 3H, OCH_3_), 6.95 (d, *J* = 3.66 Hz, 1H, FuH), 7.10 (td, *J* = 7.50, 0.73 Hz, 1H, ArH),7.20–7.23 (m, 1H, ArH), 7.39 (d, *J* = 3.63 Hz, 1H, FuH), 7.55–7.58 (m, 1H, ArH), 7.68–7.73 (m, 2H, ArH + ArH-Fu), 7.81–7.86 (m, 1H, ArH-Fu), 8.00–8.03 (m, 2H, ArH-Fu), 10.00 (s, 1H, NHCO), 10.50 (s, 1H, CONH), 10.63 (s, 1H, CONHCO); IR *ν*: 3358.1, 3227.9, 3124.2, 1715.7, 1672.0, 1598.8, 1528.0, 1478.3, 1364.7, 1292.9, 1248.9, 1225.1, 1175.6, 1107.8, 1038.0, 1015.9 cm^-1^; Anal. calcd.(%) for C_20_H_16_N_4_O_7_: C 56.61, H 3.80, N 13.20; found C 56.76, H 4.03, N 13.07.

*N-3-methylbenzoyl-N′-[5-(2′-chlorophenyl)-2-furoyl] semicarbazide* (**E10**): Light yellow powdery crystals: yield 81.2%, m.p. 209–210 °C. ^1^H-NMR *δ*: 2.39 (s, 3H, CH_3_), 7.33 (d, *J* = 3.69 Hz, 1H, FuH), 7.39 (d, *J* = 3.69 Hz, 1H, FuH), 7.41–7.55 (m, 4H, 3ArH-Fu + ArH), 7.62 (dd, *J* = 7.91,0.83 Hz, 1H, ArH),7.80–7.86 (m, 2H, ArH), 8.23 (dd, *J* = 7.88, 1.70 Hz, 1H, ArH-Fu), 10.14 (s, 1H, NHCO), 10.75 (d, *J* = 1.08 Hz, 1H, CONH), 11.05 (s, 1H, CONHCO); IR *ν*: 3413.4, 3243.4, 1711.8, 1667.3, 1516.9, 1469.5, 1316.6, 1281.4, 1231.0, 1085.0, 1029.4 cm^-1^; Anal. calcd.(%) for C_19_H_14_ClN_3_O_4_: C 60.38, H 4.05, N 10.56; found C 60.56, H 4.11, N 10.55.

*N-2,6-difluorobenzoyl-N′-[5-(2′-chlorophenyl)-2-furoyl] semicarbazide* (**E11**): Light yellow powdery crystals: yield 84.3%, m.p. 192–193 °C. ^1^H-NMR *δ*: 7.22–7.28 (m, 2H, ArH), 7.33 (d, J = 3.69 Hz, 1H, FuH), 7.38 (d, *J* = 3.69 Hz, 1H, FuH), 7.41–7.55 (m, 2H, 2ArH-Fu), 7.60–7.63 (m, 2H, ArH-Fu + ArH), 8.23 (dd, *J* = 7.85, 1.70 Hz, 1H, ArH-Fu), 9.73 (s, 1H, NHCO), 10.69 (s, 1H, CONH), 11.47 (s, 1H, CONHCO); IR *ν*: 3406.3, 3271.5, 3163.4, 1728.0, 1700.1, 1673.5, 1627.1, 1593.0, 1549.9, 1490.9, 1468.0, 1317.4, 1283.9, 1236.7, 1029.5, 1011.8 cm^-1^; Anal. calcd.(%) for C_19_H_12_ClF_2_N_3_O_4_: C 54.36, H 2.88, N 10.01; found C 54.23, H 3.08, N 10.06.

*N-2-methoxybenzoyl-N′-[5-(2′-chlorophenyl)-2-furoyl] semicarbazide* (**E12**): White powdery crystals: yield 84.3%, m.p. 190–191 °C. ^1^H-NMR *δ*: 3.88 (s, 3H, OCH_3_), 7.10 (td, *J* = 7.51, 0.81 Hz, 1H, ArH), 7.20–7.23 (m, 1H, ArH), 7.33–7.63 (m, 6H, 2FuH + 3ArH-Fu + ArH), 7.69–7.71 (m, 1H, ArH), 8.23 (dd, *J* = 7.82, 1.58 Hz, 1H, ArH-Fu), 10.02 (s, 1H, NHCO), 10.52 (d, *J* = 1.08 Hz, 1H, CONH), 10.72 (s, 1H, CONHCO); IR *ν*: 3362.4, 3226.6, 1715.6, 1669.5, 1649.9, 1598.6, 1521.2, 1479.2, 1317.9, 1292.0, 1248.7, 1225.8, 1175.3, 1016.6 cm^-1^; Anal. calcd.(%) for C_20_H_16_ClN_3_O_5_: C 58.05, H 3.90, N 10.15; found C 57.70, H 3.67, N 10.59.

*N-2,6-dichlorobenzoyl-N′-[5-(2′,4′-difluorophenyl)-2-furoyl] semicarbazide* (**E13**): Light yellow powdery crystals: yield 78.6%, m.p. 224–225 °C. ^1^H-NMR *δ*: 6.98 (brs, 1H, FuH), 7.30–7.35 (m, 2H, FuH + ArH-Fu), 7.44–7.61 (m, 4H, ArH-Fu + 3ArH), 8.26–8.31 (m, 1H, ArH-Fu), 9.85 (s, 1H, NHCO), 10.73 (s, 1H, CONH), 11.54(s, 1H, CONHCO); IR *ν*: 3421.2, 3265.8, 3158.9, 2962.2, 1739.3, 1691.4, 1665.6, 1597.3, 1535.3, 1513.6, 1483.3, 1432.0, 1314.6, 1272.2, 1225.5, 1147.4, 1103.9, 1033.7 cm^-1^; Anal. calcd.(%) for C_19_H_11_Cl_2_F_2_N_3_O_4_: C 54.36, H 2.88, N 10.01; found C 54.26, H 2.87, N 10.18.

*N-2-chlorobenzoyl-N′-[5-(2′,4′-difluorophenyl)-2-furoyl] semicarbazide* (**E14**): Light yellow powdery crystals: yield 86.2%, m.p. 216–217 °C. ^1^H-NMR *δ*: 6.98 (t, *J* = 3.72 Hz, 1H, FuH), 7.30–7.37 (m, 2H, FuH + ArH-Fu), 7.43–7.62 (m, 5H, ArH-Fu + 4ArH), 8.23–8.31 (m, 1H, ArH-Fu), 9.86 (s, 1H, NHCO), 10.73 (s, 1H, CONH), 11.27 (s, 1H, CONHCO); IR *ν*: 3411.1, 3237.3, 1729.7, 1685.3, 1658.7, 1573.2, 1526.8, 1508.7, 1482.5, 1431.9, 1312.6, 1272.0, 1241.9, 1148.5, 1104.0, 1033.9 cm^-1^; Anal. calcd.(%) for C_19_H_12_ClF_2_N_3_O_4_: C 50.24, H 2.44, N 9.25; found C 50.09, H 2.51, N 9.54.

*N-2-methoxybenzoyl-N′-[5-(4′-chlorophenyl)-2-furoyl] semicarbazide* (**E15**): White powdery crystals: yield 84.6%, m.p. 192–193 °C. ^1^H-NMR *δ*: 3.93(s, 3H, OCH_3_), 7.10(td, *J* = 7.51,0.79 Hz,1H, ArH), 7.20–7.24(m,2H, FuH+ ArH), 7.34(d, *J* = 3.57 Hz, 1H, FuH), 7.56–7.62 (m, 4H, 2ArH-Fu+2ArH), 7.99–8.02(m, 2H, ArH-Fu), 10.01 (s, 1H, NHCO), 10.52 (s, 1H, CONH), 10.70 (s, 1H, CONHCO); IR *ν*: 394.2, 3331.0, 2969.1, 1742.1, 1721.9, 1660.1, 1597.9, 1478.0, 1408.6, 1295.7, 1253.2, 1228.8, 1091.0, 1049.3, 1018.4 cm^-1^; Anal. calcd.(%) for C_20_H_16_ClN_3_O_5_: C 58.05, H 3.90, N 10.15; found C 57.76, H 3.66, N 10.42.

### 3.4. X-ray structure determination of **E2**

Compound **E2** was recrystallized from methanol to obtain colorless crystal suitable for X-ray single crystal diffraction. Cell constants at 173(2) K were determined by a least-square fit to the setting parameters of **E2** independent reflections measured on a Rigaku raxis Rapid IP Area Detector diffractometer with a graphite-monochromated Cu Kα radiation (*λ* = 0.154178 nm) and operating in the phi and scan modes. The structure was solved by direct method with SHELXS-97 [24,25] and refined by the full-matrix least squares method on F2 data using SHELXL-97 [25,26]. The empirical absorption corrections were applied to all intensity data. H atom of N—H was initially located in a difference Fourier map and was refined with the restraint *U*iso(H) = 1.2 *U*eq(N). Other H atoms were positioned geometrically and refined using a riding model, with d(C…H) = 0.093—0.097 nm and *U*iso(H) = 1.2 *U*eq(C) or 1.5 *U*eq(C-methyl). Crystallographic data for the structure have been deposited with the Cambridge Crystallographic Data Center as supplementary publication No. CCDC 769209. These data can be obtained free of charge from the CCDC via www.ccdc.cam.ac.uk/data_request/cif.

### 3.5. Biological assays

(a) The preliminary fungicidal activity *in vitro* was tested against five kinds of strains: *Phytophthora Capsici*, *Botrytis Cinerea*, *Fusarium Oxysporum*, *Rhizoctonia Solanii* and *Corynespora Cassiicola*. All the strains were conserved in the Institute of Vegetable and Flowers, Chinese Academy of Agricultural Science. Commercialized fungicides: 50% dimethomoph WP, 40% dimetachlone WP, 70% thiophanate-methyl WP, 3% jinggangmycin AS and 75% chlorothalonil WP were used as controls for the above five strains, respectively.

The fungicidal activity of the title compounds **E** against above five strains *in vitro* was evaluated using the mycelium growth rate test [27]. Both control fungicides and the title compounds were dissolved in DMF at the concentration of 50 μg mL^-1^. The culture media was obtained by mixing the solution of compounds **E** in DMF with potato dextrose agar (PDA), on which fungus cakes were placed. The blank test was made using DMF. The culture had been stored in an incubator at 24 ± 0.5 °C for 2 days. Three replicates were performed. After the mycelia grew completely, the diameters of the mycelia were measured and the inhibitory rates were calculated according to the formula:

*I* (%) = (*C*-*T*)/*C* × 100%

in which *I* stands for the inhibition (%), *C* for the diameter of mycelia in the blank control test (in mm), and *T* for the diameter of mycelia in the presence of compounds **E** (in mm). The results are shown in Table 4.

(b) Insecticidal activity against *Plutella xylostella* L., *Aphis gossypii* and *Tetranchus urticae* was evaluated using the immersion method [28]. Test solutions were made by dissolving the title compounds **E** in DMSO, adding a little 0.1% Tween-80 as the emulsifier, and then diluting to 500 μg mL^-1^ with water. The insects were raised at 27 ± 1 °C, with 12:12 h (light/dark) photoperiod and 80% relative humidity. The results were observed after 72 h and expressed by percentage death. The solvent of DMSO was set as blank control. Hexaflumuron and RH-5849 were used as insecticidal controls. Three replicates were performed. The results are shown in Table 5.

(c) Anticancer activity was screened against four different cell lines: a human promyelocytic leukemic cell line (HL-60), a human hepatocellular carcinoma cell line (Bel-7402), a human gastric carcinoma cell line (BGC-823), and a human nasopharyngeal carcinoma cell line (KB). All the title compounds were dissolved in DMSO. The four types of cell line were grown and maintained in RPMI-1640 medium supplemented with 10% fetal bovine serum (FBS), penicillin (100 U mL^-1^), and streptomycin (100 μg mL^-1^) at 37 °C in humidified incubators in an atmosphere of 5% CO_2_. 

All the experiments were performed on exponentially growing cancer cells. Numbers of viable cancer cells were determined by MTT [3-(4,5-dimethylthiazol-2-yl)-2, 5-diphenyl tetrazolium bromide] [29] and SRB [30] assays. The cancer cells (1~2.5 × 10^4^ cells mL^-1^) were inoculated in 96-well culture plates (180 μL/well). After 24 h, 20 μL of culture medium containing compounds of various concentrations were added to the wells and, the cells were incubated for 48 h. 20 μL of RPMI-1640 medium was added to the control cells. HL-60 cells were assayed by MTT, and the Bel-7402, BGC-823 and KB cells were assayed by SRB. The absorbance of each well was measured using a microculture plate reader at 570 nm (MTT) and 540 nm (SRB). The inhibition rate was calculated according to the following formula:

Inhibition rate = (ODcontrol - ODtreated)/ODcontrol × 100%

Three replicates were performed. The IC_50_ values of some active target compounds were evaluated using logit analysis **[31]**. The results are shown in Table 6.

## 4. Conclusions

In summary, a series of novel semicarbazide derivatives were synthesized by the nucleophilic addition reaction of 5-substituted aryl-2-furoyl hydrazines with benzoyl isocyanates. Compared with the lead compounds, the title compounds showed good insecticidal activity, especially towards lepidopterous insects, but exhibited lower activity against the five kinds of common plant pathogens tested. Inspiringly, the title compounds possessed obvious anticancer activity against human promyelocytic leukemic cell line (HL-60), and some of the title compounds also had activity against solid human tumor cells. The results indicated that the linkage (Y) of the lead compounds was important for the bioactivity and spectra and that modification on the urea linkage was an effective strategy to discover new pesticide and drug candidates.

## Figures and Tables

**E1**: R^1^=H, R^2^=4-Cl; **E2**: R^1^=2,6-di-F, R^2^=4-F; **E3**: R^1^=3-CH_3_, R^2^=4-Cl; **E4**: R^1^=2,6-di-F, R^2^=4-Cl; **E5**: R^1^=2-Cl, R^2^=4-Cl; **E6**: R^1^=2,6-di-Cl, R^2^=4-CH_3_; **E7**: R^1^=2,6-di-F, R^2^=4-CH_3_; **E8**: R^1^=2-Cl, R^2^=4-CH_3_; **E9**: R^1^=2-OCH_3_, R^2^=2-NO_2_; **E10**: R^1^=3-CH_3_, R^2^=2-Cl; **E11**: R^1^=2,6-di-F, R^2^=2-Cl; **E12**: R^1^=2-OCH_3_, R^2^=2-Cl; **E13**: R^1^=2,6-di-Cl, R^2^=2,4-di-F; **E14**: R^1^=2-Cl, R^2^=2,4-di-F; **E15**: R^1^=2-OCH_3_, R^2^=4-Cl.

**Table 1:** Crystal and experimental data of compound **E2**.

Empirical formula	C_19_H_12_F_3_N_3_O_4_
Formula weight	403.32
*T*	173(2) K
Wavelength	1.54178 Å
Crystal system	Triclinic
Space group	*P*1
Unit cell dimensions	a = 6.8462(12) Å, *α* = 107.173(13)° b = 11.552(2) Å, *β* = 104.549(12)° c = 11.667(2) Å, *γ* = 93.363(13)°
Volume	844.4(3) Å^3^
*Z*	2
*D_x_*	1.586 Mg/m^3^
Absorption coefficient	1.171 mm^-1^
*F* (0 0 0)	412
Crystal dimensions	0.20 x 0.13 x 0.10 mm
*θ* range for data collection	4.05 to 68.19
Completeness to *θ* = 68.19	96.7 %
Limiting indices	-8≤*h*≤8, -13≤*k*≤13,-13≤*l*≤14
Reflection collected/unique	8794/2990 [R(int) = 0.0529]
Absorption correction	Numerical
Max. and min. transmission	0.8919 and 0.7995
Data/restraints/parameters	2990 / 0 / 263
Goodness-of-fit on *F*^2^	1.088
Final *R* indices [*I* > 2*σ* (*I*)]	R_1_ = 0.0555, wR_2_ = 0.1218
2*θ*_max_	136.38° with Cu *K*_α_
(Δ*ρ*)_max_	0.301 eÅ^-^^3^
(Δ*ρ*)_min_	-0.318 eÅ^-^^3^
Program system	SHELXS-97, SHELXL-97
Structure determination	Direct method
Refinement	Full-matrix least-squares on *F*^2^
CCDC No.	769209

**Table 2:** Selected bond lengths, angels, and torsion angles of compound **E2**.

Lengths	(Å)	Angles	(°)	Torsion angles	(°)
C(4)-C(7)	1.461(4)	O(2)-C(11)-N(1)	123.0(3)	C(11)-N(1)-N(2)-C(12)	-113.9(3)
C(8)-C(11)	1.470(4)	O(2)-C(11)-C(8)	123.4(3)	C(6)-C(1)-C(2)-C(3)	2.0(5)
C(13)-C(14)	1.504(4)	O(3)-C(12)-N(3)	119.9(3)	F(1)-C(1)-C(2)-C(3)	-177.7(3)
O(2)-C(11)	1.228(3)	O(3)-C(12)-N(2)	124.9(3)	C(5)-C(4)-C(7)-O(1)	176.3(2)
O(3)-C(12)	1.224(3)	O(4)-C(13)-C(14)	120.6(3)	C(7)-O(1)-C(8)-C(9)	0.0(3)
O(4)-C(13)	1.219(3)	O(4)-C(13)-N(3)	124.2(3)	N(2)-N(1)-C(11)-O(2)	4.9(4)
N(1)-C(11)	1.363(3)	N(2)-C(12)-N(3)	115.2(2)	N(1)-N(2)-C(12)-O(3)	4.1(4)
N(2)-C(12)	1.342(3)	N(3)-C(13)-C(14)	115.2(3)	N(1)-N(2)-C(12)-N(3)	-176.2(2)
N(3)-C(13)	1.369(3)	C(13)-N(3)-C(12)	127.7(2)	C(12)-N(3)-C(13)-O(4)	6.4(4)
N(3)-C(12)	1.398(3)	C(12)-N(2)-N(1)	119.9(2)	O(4)-C(13)-C(14)-C(19)	122.7(3)
N(1)-N(2)	1.387(3)	C(11)-N(1)-N(2)	118.4(2)	C(17)-C(18)-C(19)-F(3)	179.9(3)

**Table 3:** The dihedral angles and the mean deviation of the planes in compound **E2**.

Dihedral angles (°)	
Plane I and plane II	4.2
Plane I and plane III	3.3
Plane I and plane IV	73.3
Plane I and plane V	17.5
Plane II and plane III	7.2
Plane II and plane IV	75.8
Plane II and plane V	19.9
Plane III and plane IV	70.1
Plane III and plane V	14.5
plane IV and plane V	55.9
The mean deviation of the plane (Å)	
Plane I	0.0099
Plane II	0.0009
Plane III	0.0144
Plane IV	0.0163
Plane V	0.0075

**Table 4:** Fungicidal activity of compounds **E** against five fungus species at 50 μg mL^-1^.

Compd.	Inhibitory rate (%)				
	*Phytophthora Capsici*	*Botrytis Cinerea*	*Fusarium Oxysporum*	*Rhizoctonia Solanii*	*Corynespora Cassiicola*
**E1**	1.94	13.61	7.50	0.00	2.22
**E2**	9.72	9.17	37.50	3.61	14.44
**E3**	22.50	6.11	29.17	1.39	8.33
**E4**	37.50	23.33	10.28	41.39	6.39
**E5**	23.61	54.72	32.22	0.00	8.06
**E6**	49.17	49.17	14.17	0.00	11.39
**E7**	8.89	29.17	5.83	0.00	5.83
**E8**	31.94	48.89	14.44	8.33	8.33
**E9**	10.28	28.61	28.89	0.56	10.28
**E10**	12.50	0.00	20.83	0.00	8.33
**E11**	19.44	0.00	9.72	50.00	6.94
**E12**	20.83	45.28	4.72	0.00	12.22
**E13**	13.06	4.17	8.61	0.00	19.72
**E14**	21.67	7.78	8.61	0.00	18.06
**E15**	4.17	10.56	30.28	0.00	5.00
**DMF(control)**	4.17	5.56	22.78	13.06	14.72
**fungicides^a^**	86.39 a	69.72 b	68.33 c	76.67 d	68.61 e

^a^Control fungicides: a, 50% dimethomoph WP; b, 40% dimetachlone WP; c, 70% thiophanate-methyl WP; d, 3% jinggangmycin AS; e, 75%chlorothalonil WP

**Table 5:** Insecticidal activity of compounds **E** at 500 μg mL^-1^.

Compd.	Inhibitory rate (%)		
	*Plutella xylostella* L.	*Aphis gossypii*	*Tetranchus urticae*
**E1**	15.0	20.0	0
**E2**	32.0	20.0	0
**E3**	33.0	29.4	0
**E4**	30.0	17.1	0
**E5**	25.0	23.1	0
**E6**	20.0	12.9	0
**E7**	14.0	27.3	0
**E8**	24.0	32.7	0
**E9**	0	18.5	0
**E10**	95.0	15.4	0
**E11**	45.0	23.2	0
**E12**	16.0	19.2	0
**E13**	10.0	38.1	0
**E14**	0	24.5	0
**E15**	85.0	14.8	0
CK	0	2.4	0
Hexaflumuron	100	22.6	100
RH-5849	100	22.1	100

NT: not detected.

**Table 6:** The IC_50_ values of antitumor activity of compounds **E**.

Compd.	IC_50_ (mM)			
	HL-60	Bel-7402	BGC-823	KB
**E1**	1.56	-	-	-
**E2**	1.26	-	-	-
**E3**	-	-	-	-
**E4**	-	-	-	-
**E5**	0.13	-	-	-
**E6**	-	-	-	-
**E7**	11.99	5.39	-	-
**E8**	-	-	-	-
**E9**	5.39	-	-	-
**E10**	-	-	-	-
**E11**	0.44	11.02	1.42	-
**E12**	2.93	-	-	-
**E13**	0.18	-	59.46	-
**E14**	2.63	-	-	-
**E15**	0.14	0.36	-	0.09

- means IC_50_ > 100 mM.

## References

[1] Cohen E. (2001). Chitin synthesis and inhibition: A revisit. Pest Manag. Sci..

[2] Ruiz-Herrera J., San-Blas G. (2003). Chitin synthesis as a target for antifungal drugs. Curr. Drug Targets Infect. Disord..

[3] Wellinga K., Mulder R., Dallen J.V. (1973). Synthesis and laboratory evaluation of 1-(2,6-disubtituted benzoyl)-3-phenylureas, a new class of insecticides. I. 1-(2,5-Dichlorobenzoyl)-3- phenylureas. J. Agric. Food Chem..

[4] Oberlander H., Silhacek D.L., Ishaaya I., Degheele D. (1998). New Perspectives on the Mode of Action of Benzoylphenyl Urea Insecticides. Insecticides with Novel Modes of Action: Mechanism and Application.

[5] Oberlander H., Silhacek D.L. (1998). Mode of action of insect growth regulators in Lepidopteran tissue culture. Pestic. Sci..

[6] Qian X.H. (1999). Quantitative studies on structure-activity relationship of sulfonylurea and benzoylphenylurea type pesticides and their substituents’ bioisosterism using synthons’ activity contribution. J. Agric. Food Chem..

[7] Nakagawa Y., Kitahara K., Nishioka T., Iwamura H., Fujita T. (1984). Quantitative structure- activity studies of benzoylphenylurea larvicides: I. Effect of substituents at aniline moiety against Chilo suppressalis walker. Pestic. Biochem. Physiol..

[8] Tecle B., Ruzo L.O., Casida J.E. (1988). Radiosynthesis of [benzoyl-3,4,5-3*H*]diflubenzuron by a route applicable to other high-potency Insect Growth Regulators. J. Agric. Food Chem..

[9] Hashizume B. (1988). Atabron 5E, a new IGR insecticide (chlorfluazuron). Jpn. Pestic. Inf..

[B10-molecules-15-04267] 10.Sbragia, R.J.; Johnson, G.W.; Karr, L.L.; Edwards, J.M.; Schneider, B.M. Preparation of benzoylphenylurea insecticides to control cockroaches, ants, fleas, and termites. WO 9819542, 1998; [*Chem. Abstr.* **1998**, *129*, 13497].

[11] Yoon C., Yang J.O., Kang S.H., Kim G.H. (2008). Insecticidal properties of bistrifluron against sycamore lace bug, Corythucha ciliata (Hemiptera:Tingidae). J. Pestic. Sci..

[12] Grosscurt A.C., Van H.R., Wellinga K. (1979). 1-Phenylcarbamoyl-2-pyrazolines, a new class of insecticides. 3. Synthesis and insecticidal properties of 3,4-diphenyl-1-phenylcarbamoyl-2-pyrazolines. J. Agric. Food Chem..

[13] Chen L., Wang Q.M., Huang R.Q., Mao C.H., Shang J., Bi F.C. (2005). Synthesis and insecticidal evaluation of propesticides of benzoylphenylureas. J. Agric. Food Chem..

[14] Chen L., Huang Z.Q., Wang Q.M., Shang J., Huang R.Q., Bi F.C. (2007). Insecticidal benzoylphenylurea-S-carbamate: A new propesticide with two effects of both benzoylphenylureas and carbamates. J. Agric. Food Chem..

[15] Sun R.F., Zheng Y.L., Chen L., Li Y.Q., Li Q.S., Song H.B., Huang R.Q., Bi F.C., Wang Q.M. (2009). Design, synthesis and insecticidal activities of new *N*-benzoyl-*N*-phenyl-*N*- sulfenylureas. J. Agric. Food Chem..

[16] Yang X.L., Ling Y., Wang D.Q., Chen F.H. (2002). The synthesis and biological activity of *N*-phenyl-*N*-(5-phenyl-2-furoyl) ureas. Chin. J. Synth. Chem..

[17] Yang X.L., Wang D.Q., Chen F.H., Ling Y., Zhang Z.N., Shang Z.Z. (1997). Studies on the design, synthesis and biological activity of a novel type of acylurea compound. Chem. J. Chin. Univ..

[18] Yang X.L., Wang D.Q., Chen F.H., Ling Y., Zhang Z.N. (1998). The synthesis and larvicidal activity of *N*-aroyl-*N*-(5-aryl-2-furoyl) ureas. Pestic. Sci..

[19] Li Y., Li B.J., Ling Y., Miao H.J., Shi Y.X., Yang X.L. (2010). Synthesis and Fungicidal Activity of Aryl Carbamic Acid-5-aryl-2-furanmethyl Ester. J. Agric. Food Chem..

[20] Ren S.J., Wang R.B., Komatsu K., Bonaz-Krause P., Zyrianov Y., McKenna C.E., Csipke C., Tokes Z.A., Lien E.J. (2002). Synthesis, biological evaluation, and quantitative structure-activity relationship analysis of new Schiff bases of hydroxysemicarbazide as potential antitumor agents. J. Med. Chem..

[21] Nishat N., Ahamad T., Alshehri S.M., Parveen S. (2010). Synthesis, characterization, and biocide properties of semicarbazide-formaldehyde resin and its polymer metal complexes. Eur. J. Med. Chem..

[22] Speziale A.J., Smith L.R. (1962). New and convenient synthesis of acyl isocyanates. J. Org. Chem..

[23] Cui Z.N., Huang J., Li Y., Ling Y., Yang X.L., Chen F.H. (2008). Synthesis and bioactivity of novel *N*,*N′*-diacylhydrazine derivatives containing furan(I). Chin. J. Chem..

[B24-molecules-15-04267] 24.Bruker (1998) SMART. Bruker AXS Inc.: Madsion, WI, USA, 1998.

[B25-molecules-15-04267] 25.Sheldrick, G. M. (1997) SHELXS97 and SHELXL 97. University of Göttingen: Germany, 1997.

[B26-molecules-15-04267] 26.Bruker (1999) SAINT and SHELXTL. Bruker AXS Inc.: Madison, WI, USA, 1999.

[27] Chen N.C. (1991). Bioassay of Pesticides.

[28] Chen N.C. (1991). Bioassay of Pesticides.

[29] Denizot F., Lang R. (1986). Rapid colorimetric assay for cell growth and survival. Modifications to the tetrazolium dye procedure giving improved sensitivity and reliability. J. Immunol. Methods.

[30] Monks A., Scudiero D., Skehan P., Shoemaker R., Paull K., Vistica D., Hose C., Langley J., Cronise P., Vaigro-Wolff A., Gray-Goodrich M., Campbell, H., Mayo J., Boyd M. (1991). Feasibility of a high-flux anticancer drug screen using a diverse panel of cultured human tumor cell lines. J. Natl. Cancer Inst..

[31] Berkson J. (1944). Application of the logistic function to bio-assay. J. Am. Statistical Assoc..

